# Notes and Notices

**Published:** 1885-05

**Authors:** 


### NOTES AND NOTICES.

ANNUAL MEETING OF THE HOMŒOPATHIC MEDICAL SOCIETY.—The Homœopathic Medical Society of Ohio will convene at Cincinnati, on May 13th and 14th, 1885. The sessions will be held at the Pulte Medical College, with headquarters at the Palace Hotel (rates, $2.00 per day).

H. E. BEEBE, *Secretary*.

DR. JOHN V. MILLER, formerly of Newark, N. J., has removed to New York city, taking charge of the practice of the late Dr. Constantine Lippe. Dr. Miller will be succeeded at Newark by Dr. Harlyn Hitchcock, a recent convert from allopathic empiricism. As both of these gentlemen are true homœopaths and able practitioners, both New York and Newark will be well cared for.

A NEW POTENTIZER.—Our friend, Dr. R. B. Johnstone, has invented a new instrument for potentizing homœopathic medicines. He hopes to exhibit it at the coming meeting of the I. H. A. Messrs. Fincke, Swan, Skinner, and Foote, etc., will have to look out, else they will lose their laurels as inventors!

DR. LILIENTHAL, the veteran editor, writes us he has cast aside all his burdens, and will soon hie himself away to foreign shores. We are sure no one wishes him a pleasant trip with more heartiness than does the HOMŒOPATHIC PHYSICIAN. *Bon voyage.*

DR. BURNETT.—The numerous readers of the sprightly and interesting *Homœopathic World* will be sorry to learn that J. Compton Burnett will no longer fill its editorial chair. Dr. Clarke, his successor, will have to show his best work to avoid unfavorable comparison. Yet we think he will prove no mean competitor.

HOMŒOPATHIC CONGRESS.—The next International Homœopathic Congress will meet at Brussels, in 1886, at a time to be decided upon later. Those desiring further information should write either Dr. Richard Hughes (Brighton, England), permanent secretary, or to Dr. Martiny, Brussels, Belgium. The value of these international meetings cannot be questioned.

